# Salivary biomarkers present in patients with periodontitis without clinical distinction: findings from a meta-analysis

**DOI:** 10.4317/medoral.25876

**Published:** 2023-04-07

**Authors:** Paulo Roberto Carneiro Gomes, Maria Débora Rodrigues da Rocha, John Arlley Sousa Pinho de Lira, Francisco Alex da Rocha Coelho, Even Herlany Pereira Alves, Hélio Mateus Silva Nascimento, Samara Marques de Oliveira, Rubens Renato de Sousa Carmo, Havila Torres Araújo, Felipe Rodolfo Pereira da Silva, Daniel Fernando Pereira Vasconcelos

**Affiliations:** 1 Laboratory of Histological Analysis and Preparation (LAPHis), Parnaiba Delta Federal University (UFDPar), Parnaiba, Brazil; 2 Laboratory of Pain neuromodulation and sensorimotor performance, (LANDS), Parnaiba Delta Federal University (UFDPar), Parnaíba, Brazil; 3 Parnaiba Delta Federal University; 4 Laboratory of Infectious Diseases (LADIC), Parnaiba Delta Federal University (UFDPar), Parnaíba, Brazil; 5 Advanced Study Group in Mycology, Parnaiba Delta Federal University (UFDPar), Parnaíba, Brazil; 6 Medicine College, Altamira University Campus, Federal University of Pará (UFPA), Altamira, PA, Brazil

## Abstract

**Background:**

A new classification for periodontitis has been adopted in clinical practice. However, there are still discussions regarding this new classification and difficulties in its adoption, both by professionals and researchers. Thus, this study aimed to evaluate which salivary biomarkers are present in periodontitis, following the new classification of periodontal diseases through meta-analysis.

**Material and Methods:**

A literature search was carried out in the scientific databases: PubMed, Scielo and Google scholar to select studies. The selection of studies was followed by two authors upon reading of the title, abstract and full text. The necessary data were collected and statistical analyses were performed using the Review Manager statistical software version 5.4, with calculation of Mean Difference, heterogeneity (I²) and funnel plot with *P* < 0.05.

**Results:**

After following the selection criteria, 9 articles were selected for comparison. The studies address the presence of biomarkers in the saliva of patients with periodontitis and their possible use in the monitoring and diagnosis of the disease. For the meta-analytic comparison, a sample size of 1,983 individuals was used. Statistical analyses showed that nitric oxide, IL-6, IL-1B and osteoprotegerin are substances that are significantly present in patients with periodontitis (*P* < 0.05).

**Conclusions:**

IL-6, nitric oxide, IL-1B, TNF-α and osteoprotegerin are among the most present biomarkers in patients with periodontitis, and may be used in the future as a monitoring of periodontal disease. The present study also revealed that there was no statistically significant difference in the concentration of these biomarkers for clinical distinction from periodontitis.

** Key words:**Periodontal disease, diagnosis, spittle.

## Introduction

Periodontitis is a multifactorial disease caused by the formation of a bacterial biofilm that primarily affects the tissue supporting the teeth, the periodontium. The epidemiology of the disease remains increased worldwide. In the Norwegian population, the prevalence of the disease in advanced stages was found to be 17.6% from 4,863 participants (1). In another study (2), the data showed an increased global prevalence of periodontitis in which the Western Sub-Saharan Africa carried the heaviest burden of periodontitis, whereas the nation with the highest periodontitis burden was Gambia.

Regarding periodontal disease, a new classification has been adopted for periodontitis in order to minimize mistakes in clinical practice. What was named as "chronic periodontitis" and "aggressive periodontitis" has been categorized, since 2017, only as "periodontitis" (3). In this disease, specific anaerobic gram-negative bacteria are present, such as: *Porphyromonas *gingivalis**, *Treponema denticola*, *Rothia* dentocariosa, *Fusobacterium animalis*, *Streptococcus oralis*, *Veillonella dispar*,c*Atopobium parvulum*, *Tannerella forsythia* and *Aggregatibacter actinomycetemcomitans*. These microorganisms accumulate in the periodontium region and cause an inflammatory response resulting in the release of various cytokines, enzymes and inflammatory cells (4).

In the course of periodontitis, chemokines such as chemokine (C-X-C motif) ligand 1 (CXCL-1), C-X-C motif chemokine ligand 8 (CXCL-8), C-C motif chemokine ligand 5 CCL-5 and cytokines such as tumor necrosis factor alpha (TNF-α), interleukin (IL)-1β, IL-6 are present in the inflammatory process of periodontitis and are responsible for recruiting T cells, neutrophil chemotaxis and increased permeability of small blood vessels (5)

Saliva is produced by the salivary glands, being one of the main constituents of the digestion process as it is rich in enzymes that act in the breakdown of lipids and starches (6). The components of saliva are derived from the blood and are divided into inorganics, such as sodium, potassium, calcium, chloride and bicarbonate, while the organic ones are glucose, enzymes, proteins and urea (7). Research suggests the potential of this secretion within clinical practice, and it may be useful for the diagnosis and monitoring of comorbidities such as diabetes (8), tumor and intestinal diseases (9). This reality has not been different in periodontitis and the literature points to several biomarkers present in the salivary fluid that can be used for the early diagnosis and follow-up of periodontal disease (10).

However, the data on biomarkers in the saliva that help the periodontitis diagnosis are contradictory and require a better evaluation. Therefore, the aim of this study was to carry out a meta-analysis to investigate salivary biomarkers present in periodontitis.

## Material and Methods

We have followed the Preferred Reporting Items for Systematic Reviews and Meta-analyses (PRISMA) for the delineation of this study (11).

- Study design

This study is a systematic evaluation with meta-analysis that focused on the association between salivary biomarker levels and periodontitis. As a meta-analysis, this study does not require approval by the Ethics committee.

- Inclusion criteria

Studies that addressed salivary biomarkers in humans as a periodontitis-related result were included. For statistical analysis, only studies that provided the sufficient data for the calculations were included.

- Exclusion criteria

 We excluded studies that addressed the relationship of periodontitis with smokers, alcohol users and that evaluated salivary biomarkers associated with periodontitis in patients with genetic diseases or other disorders. We did not consider research that addressed drug users and who were undergoing treatment for periodontitis. Letters, bibliographic reviews, book chapters and personal opinions were not considered to compose this study. Research with insufficient data to compose the meta-analysis were excluded.

- Search Strategy

Two authors have performed a comprehensive search in literature for studies that addressed the relationship between salivary biomarkers from the host and periodontitis. The databases used were: PubMed, Scielo and Google Scholar with combined descriptors (salivary biomarkers, inflammation or infection, periodontal diseases or periodontitis). After the search, the articles were analyzed and selected following reading in two phases: (1) title and abstract; (2) full text. When the studies were not related to the purpose of this research in its title and abstract, the full text was not read and the article was excluded.

- Data collection process

Two investigators reviewed all studies and collected data that composed the Table with the main information of the studies (First author, country, study model, the salivary biomarker analyzed, total of participants and data for the statistical analysis (mean and standard deviation values)). Some studies did not provide mean and SD data. Instead, they brought data on median and quartile. Therefore, we performed the conversion of these data as described by Hozo and collaborators (12).

- Statistical Analysis

The statistical analysis was processed with the Review Manager 5.4 statistical software (Cochrane Software Review Manager). For comparison, we used continuous data and mean differences in the program. When the value of heterogeneity (I2) was not significant (<50%), the Fixed-effect model was used to estimate the mean difference (MD) with 95% of Confidence Intervals (CI). On the contrary, when the value of I2 was significant (>50%), the random effect was used for statistical calculation. We consider in both cases the value of *P* < 0.05 as statically significant. In order to assess publication bias, Begg's statistical tests and Egger's linear regression test (with *P* <0.05) were used. The asymmetry in the funnel plot for publication bias was also observed to confirm the results in Begg´s and Egger´s tests. Data from the included studies were dichotomous and expressed as OR with 95% confidence interval (CI) to verify the relationship between salivary biomarkers and periodontitis.

## Results

- Study selection

**Figure 1:**
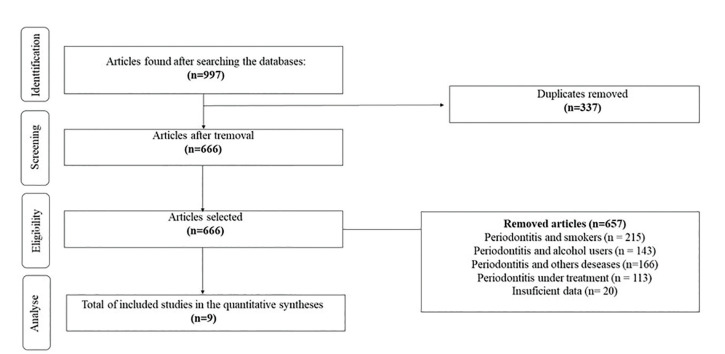
Flow diagram for identification, screening, selection and inclusion of the studies in this meta-analysis.

After the electronic search, we selected 9 studies from 997. The selection of the studies followed two steps of a previous analysis, as already detailed in the methodology. After the application of phase 1,453 remained, which were directed to phase 2; of these, 9 were used for the present study. The flowchart in Fig. 1 details the entire process of choosing the studies.

- Study characteristics

The selected studies have been published in the past 16 years and were carried out in 4 different countries: Brazil (13), India (14-16), United States of America (17-20) and Sweden (21).

The research related the main biomarkers present in saliva and the potential to use them as a diagnosis. Most of the biomarkers cited are proteins and glycoproteins that act in the inflammatory process which the data were obtained from 1,983 individuals (Table 1). The data brings results on levels of immunoglobulin A (IgA) in 134 participants (15,16,21), IL-1B in 390 patients (15,17-20), matrix metalproteinase 8 (MMP-8) in 272 participants (16-19), TNF in 165 participants (17,19), matrix metalproteinase 9 (MMP-9) in 85 participants (17,19), IL-6 in 165 subjects (17,19), IL-10 in 85 participants (19), IL-13 in 85 patients (19), IL-4 in 85 patients (19) and IL-2 in 85 participants (19). Some studies have reported nitric oxide levels in 120 participants (13,14), calprotectin in 85 subjects (19), type I collagen pyridinoline-crosslinked carboxyterminal telopeptide (ICTP) in 85 patients (19), and osteoprogerin in 142 participants (8,19). Some studies have reported the levels of nitric oxide in 120 participants (13,14), calprotectin in 85 participants (19), pyridinoline cross-linked carboxyterminal telopeptide of type I collagen in 85 participants (ICTP) (19) and osteoprogerin, also in 85 participants (8,19). A summary of the descriptive characteristics of the studies is presented in Table 1.

**Table 1:**
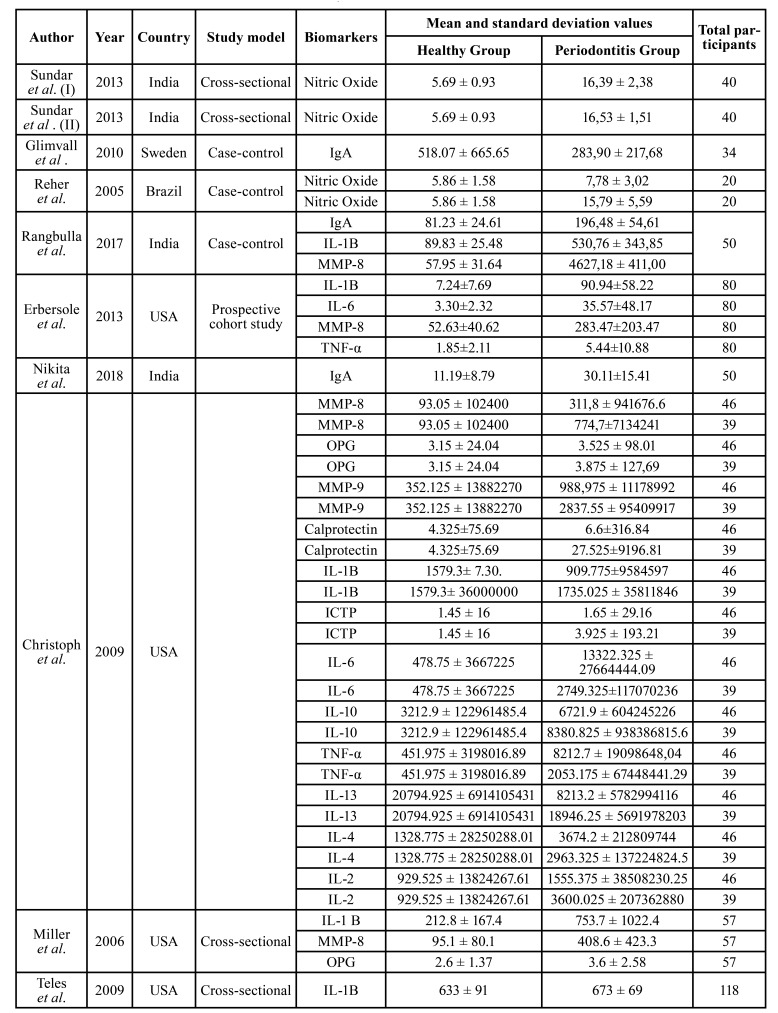
Characteristics of included studies in this meta-analysis.

- Meta-analysis

**Figure 2:**
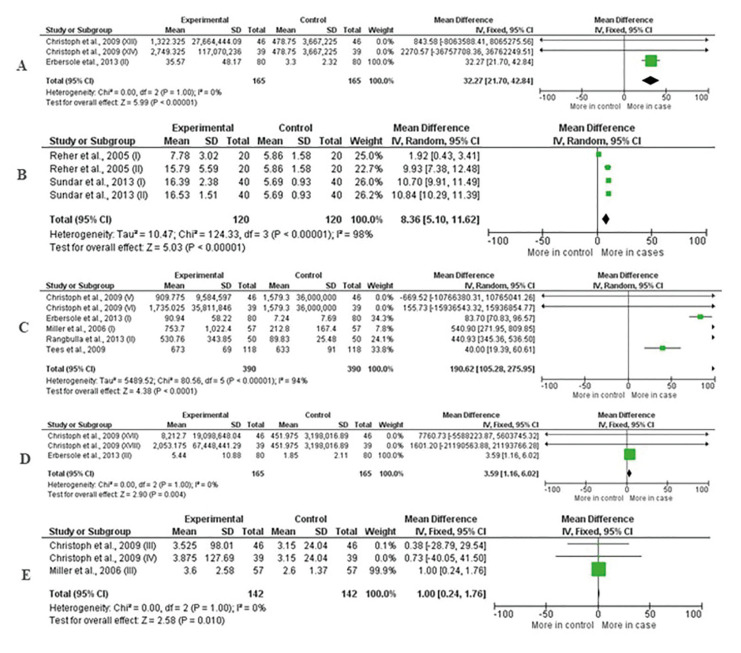
Forest plot of the comparison of the concentration of salivary biomarkers between the healthy control group and the periodontitis group. In A, IL-6; B, nitric oxide; C, IL-1B; D, TNF and E, osteoprotegerin.

The statistical analysis shows that IL-6 and nitric oxide were the biomarkers that showed the greatest value of significant association with periodontitis (*P* < 0.00001), accompanied by IL-1B (*P* < 0.0001), TNF-α (*P* = 0.004) and osteoprotegerin (*P* = 0.01). When comparing the data, one could observe that high values of these biomarkers are associated with patients with periodontitis, because the mean differences were: IL-6: 37.27 (CI: 21.70, 42.84); Nitric oxide: 8.36 (CI: 5.10, 11.62); IL-1B: 190.62 (CI: 105.28, 275.95), TNF-α: 3.59 (CI: 1.16, 6.02) and osteoprotegerin (CI: 0.24, 1.76). These dates are presented in Fig. 2.

**Figure 3:**
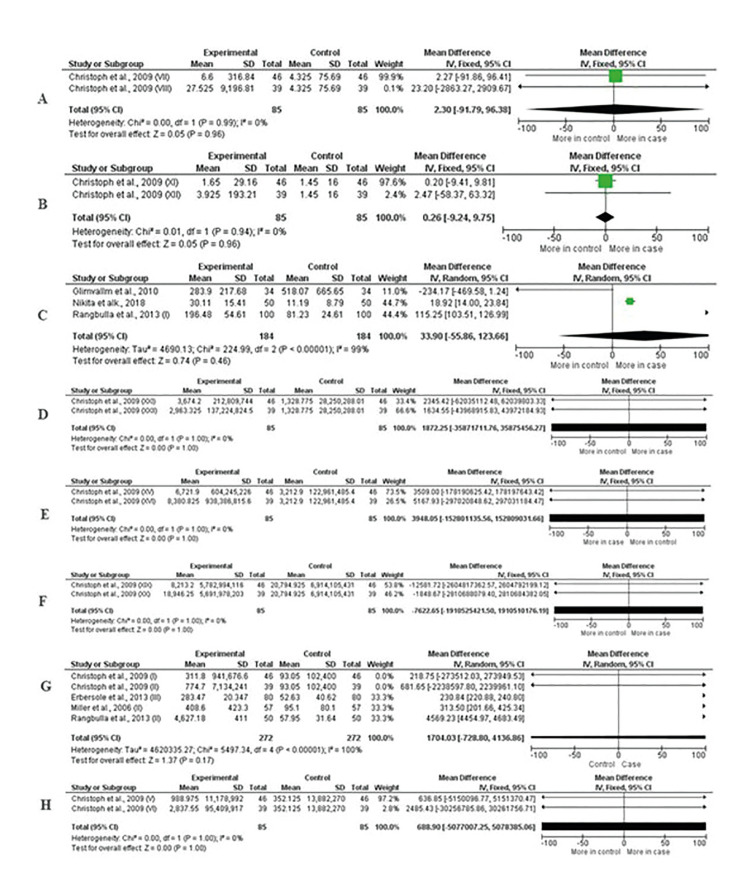
Forest plot of the comparison of the concentration of salivary biomarkers between the healthy control group and the periodontitis group. We can observe the non-significance in the comparison. In A, calprotectin; B, ICTP; C, IgA; D, IL-4; E, IL-10; F, IL-13; G, MMP-8; H, MMP-9.

The other biomarkers were not statistically significant when related to periodontitis: calprotectin (MD = 2.30, CI: -91.79, 96.38, *P* = 0.96), ICTP (MD = 0.26, CI: -9.24, 9.75, *P* = 0.96), IgA (MD = 33.90, CI: -55.86, 123.66, *P* = 0.46), IL-4 (MD = 187.25, CI: -35871711.76, 35875456.27, *P* = 1.00), IL-10 (MD = 3948.05, CI: -152801135.56, 152809031.66, *P* = 1.00), IL-13 (MD = -7622.65, CI: -1910525421.50, 1910510176.19, *P* = 1.00), MMP-8 (MD = 1704.03, CI: -728.80, 4136.86, *P* = 0.17), MMP-9 (MD = 688.90, CI: -5077007.25, 5078385.06, *P* = 1.00). All data are shown in the Fig. 3.

- Risk of Bias

**Figure 4:**
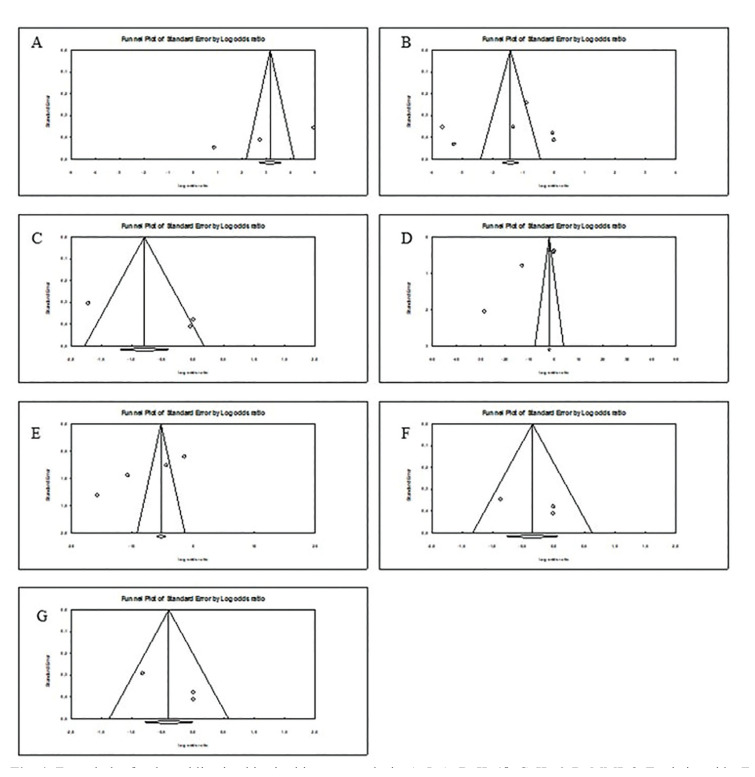
Funnel plot for the publication bias in this meta-analysis. A, IgA; B, IL-1β; C, IL-6; D, MMP-8; E, nitric oxide; F, oesteoprotegerin; G, TNF-α.

The sensitive analysis showed that most studies did not change the pooled OR value. When observing the values of Begg´s test and Egger's linear regression test (Table 2), no evidence of publication bias was found for the biomarkers IL1β, IL-6, IgA, osteoprotegerin and TNF-α, validating the results presented. However, when analyzing Egger's values for MMP-8 and nitric oxide, a possible publication bias is noticeable, given the variables that may have interfered in the results of the included studies. These data are reinforced by the Funnel plots in Fig. 4. The publication bias analyses for calprotectin, IL-4, IL-10, IL-13, ICTP and MMP-9, could not be performed due to insufficient data in the studies.

**Table 2:**
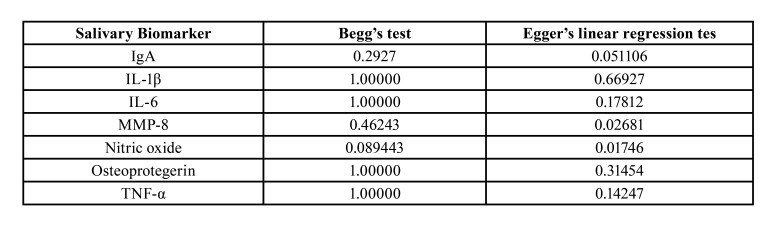
P-Values for Begg’s Test and Egger’s Linear Regression Test in this Current Meta-Analysis.

## Discussion

Salivary biomarkers have been a relevant tool in clinical practice, as they have helped in the diagnosis, monitoring and medical evaluation of diseases. This scenario is not distant in periodontal disease, since the literature describes several salivary biomarkers, derived or not from the host, that are present in periodontitis and that may serve as a diagnostic resource for this disease (10).

Although some meta-analyses have focused on determining the presence of salivary biomarkers in the different clinical classifications of the disease (15), our study aimed to evaluate the biomarkers present in the saliva of the host in periodontitis, without any clinical distinction of the disease, following the new clinical classification of periodontitis.

Aggressive periodontitis stands out for the formation of deep periodontal pockets caused by aggressive attachment loss and intense bone resorption. It is considered as a low-prevalence and rapid-progression pathology. On the other hand, chronic periodontitis is a slowly progressing disease that, added to environmental factors, causes persistent damage to the soft tissues supporting teeth and is the most common form of periodontal disease (22). A new structure was adopted for the classification of the disease. In this sense, "chronic" and "aggressive" periodontitis were gathered in a single classification: periodontitis (3). Thus, we evaluated in general terms the salivary biomarkers in periodontitis, contributing with new data for this classification.

Our meta-analysis demonstrated that IL-6 is a biomarker with a significant presence in the saliva of patients with periodontitis. IL-6 consists of a pro-inflammatory cytokine that acts in the differentiation of cells, such as B lymphocytes and in the manifestation of several proteins that act in the inflammatory process (23). Under physiological conditions, this interleukin is found at low levels in the body. However, in inflammatory processes there is an increase in its concentration, a fact that is observed in individuals with periodontitis. This increase in IL-6 is strongly correlated with the presence of C-reactive protein and may be closely linked to oral parameters of patients with periodontitis, such as increased probing pocket depth and number of teeth (24).

The presence of IL-1β is well described in patients with periodontitis when compared to healthy individuals (*P* <0.0001), but it does not show a significant difference when comparing patients with chronic and aggressive periodontitis (*P* =1.00). As the IL-6, IL-1B is a pro-inflammatory cytokine that acts on the expansion of the immune response, specifically on the differentiation of lymphocytes and the induction of adhesion molecules. In the inflammatory process of periodontitis, this cytokine seems to be closely linked to the bone resorption process, as it has osteoclast-activating activity (25). TNF-α, a cytokine secreted by macrophages, has activity like IL-1B and is linked to the tissue destruction process caused by periodontal disease and its presence in saliva is more associated with patients with periodontitis than healthy patients (*P* =0.0004). However, there is no statistically significant difference when comparing chronic periodontitis and aggressive periodontitis (19).

Another biomarker present in our results is the nitric oxide. This molecule plays important physiological roles, such as blood pressure regulation, platelet activation and immunoregulatory action (13). This free radical can be found in the saliva of patients with periodontitis (*P* < 0.00001) by the action of anaerobic facultative bacteria present in the mouth, which convert salivary nitrate into nitrite, and the latter induces the gastric production of nitric oxide (26). Results from a previous meta-analysis showed that there is no statistically significant difference in the nitric oxide concentrations of chronic periodontitis and aggressive periodontitis (14), corroborating with the new classification for the disease.

Osteoprotegerin is a protein synthesized by cells that participate in the formation of the organic portion of the bone matrix, the osteoblasts. It acts against bone resorption through a competitive process that binds by affinity to RANK ligand (RANKL), transmembrane proteins that activate osteoclasts when they bind to RANK. This interaction prevents the association of RANKL to RANK, which consequently blocks the production of osteoclasts (27). Our meta-analysis demonstrated that high levels of osteoprotegerin in saliva are more linked to the experimental group than to the healthy control group and that there is no significant difference considering the two clinical classifications of the disease. Here, we evaluated other biomarkers present in saliva, but which did not show statistically significant differences when comparing the periodontitis group to the healthy group.

IgA is a protein that is part of the antibody class and is present in the mucosa, playing an important role in immunological protection (28). Regarding the presence of IgA in saliva, no significant difference in concentration was observed in the comparison between groups. The same was observed in the concentrations of MMP-8, a collagen matrix degradation enzyme present in the early stages of periodontitis, originating from polymorphonuclear leukocytes (29). In our study, we did not find a difference in the concentration of this enzyme in the salivary fluid between the groups. Comparing the chronic periodontitis and aggressive periodontitis data, we also observed that there was no difference in the MMP-8 concentration in the saliva of the groups (*P* = 1.00) (19).

In our comparison, we found six more biomarkers that also showed no statistically significant difference between the periodontitis group and the healthy group: IL-4, IL-10, IL-13, MMP-9, calprotectin and ICTP. However, these data need to be analyzed with caution, due to limitations in the number of studies that may restrict the statistical calculation, and that does not represent a clinical non-significance.

The IL’s 4, 10 and 13 are anti-inflammatory cytokines secreted by T helper 2 (Th2) CD4+ T lymphocytes that act in the immune response inducing B cell proliferation and differentiation through biochemical signals and antibody production (30). Previous studies showed that IL-4 acts to improve periodontal disease through its regulatory potential against IL-1 and TNF-α and its ability to induce the death of activated macrophages. Furthermore, the highest concentrations of this interleukin are related to the periodontal healthy group, demonstrating a depletion of this anti-inflammatory in patients with periodontitis (31).

IL-10 is identified, together with IL-4, as an inhibitor of metalloproteinase and osteoprotegerin activity in periodontitis, although our study shows that there is no significant difference in the concentration of this cytokine in saliva between the periodontitis and healthy control groups, the literature indicates that the highest levels of IL-10 are associated with the healthy group (32). IL-13 inhibits the destructive activity of fibroblasts in periodontitis and activates the Transforming growth factor beta (TGF-β) (33), which has the function of stimulating cell growth, playing an important role in connective tissue remodeling. High levels of this biomarker are related to the periodontal healthy group (19).

MMP-9 is an enzyme that is present in several biological processes, acting in the breakdown of the extracellular matrix in processes such as bone formation and embryonic development (34,35). This metalloproteinase has already been investigated for its biomarker potential in the diagnosis and prognosis of periodontitis (36). High concentrations of MMP-9 have been correlated with high gingival bleeding and intense dental motility and, together with MMP-2, it is responsible for a greater degradation of extracellular matrix constituents in periodontitis.

Calprotectin, also known as S100A8 and S100A9 protein complex, is a heterodimer that can be found in leukocytes, such as neutrophils and monocytes, and has antibacterial activity (37). This compound has a pro-inflammatory role and has been widely used as a marker of inflammation, because during this process, this protein is expelled by myeloid cells. Altered concentrations of this protein have been present in patients with periodontitis accompanied by other mediators of inflammation, such as prostaglandin E2 and interleukin-1β. Previous studies have reported that the presence of lipopolysaccharides (LPS) from the membrane of bacteria such as *Porphyromonas *gingivalis** and *Fusobacterium nucleatum* are able to stimulate the release of calprotectin by neutrophils. In addition to calprotectin exhibiting antimicrobial activity, its expression is believed to enhance the action of pro-inflammatory cytokines such as TNF-α and IL-6 (38).

ICTP is an important marker released after collagen degradation and osteoclastic bone resorption as a result of the modification of collagen molecules after the translation process. It is found to be elevated in several metabolic bone diseases (39). The literature reports the presence of this biomarker in the saliva of patients with periodontitis, arising from the alveolar bone resorption that the disease causes (40). In the comparison performed by our meta-analysis, we showed that there was no statistically significant difference between the healthy group and the periodontitis group.

This is the first meta-analytic study to evaluate the levels of salivary biomarkers under the new classification for periodontitis. We bring relevant information for this issue. However, some important limitations should be noted and discussed. First, the high level of methodological heterogeneity in this study may represent an important bias in the evaluation. Further studies should focus on following a methodological pattern to avoid this bias. Second, the absence of important data on the included studies did not allow a complete evaluation on the risk of bias in this meta-analysis. The authors should include all relevant data in their future studies to prevent the lack of possible information on the meta-analysis.

In conclusion, our results demonstrated, through meta-analytic methods with 1,983 participants from 9 studies, that IL-6, nitric oxide, IL-1B, TNF-α and osteoprotegerin are among the most present biomarkers in patients with periodontitis. Furthermore, this study clarified that there is no statistically significant difference when comparing the concentration of biomarkers present in saliva in the previous clinical classification of the disease. Thus, the data presented here contribute to support the understanding of biomarkers levels in patients with periodontitis.
